# Cerebral Tissue Regional Oxygen Saturation as a Valuable Monitoring Parameter in Pediatric Patients Undergoing Extracorporeal Membrane Oxygenation

**DOI:** 10.3389/fped.2021.669683

**Published:** 2021-06-04

**Authors:** Song Chen, Fang Fang, Wenjun Liu, Chengjun Liu, Feng Xu

**Affiliations:** Department of Critical Care Medicine, Children's Hospital of Chongqing Medical University, National Clinical Research Center for Child Health and Disorders, Ministry of Education Key Laboratory of Child Development and Disorders, China International Science and Technology Cooperation Base of Child Development and Critical Disorders, Chongqing Key Laboratory of Pediatrics, Chongqing, China

**Keywords:** mortality, regional oxygen saturation, extracorporeal membrane oxygenation, NIRS, pediatric

## Abstract

**Objective:** Brain function monitoring technology for extracorporeal membrane oxygenation (ECMO) support has been developing quite slowly. Our objective was to explore the data distribution, variation trend, and variability of cerebral tissue regional oxygen saturation (CrSO_2_) in pediatric patients undergoing ECMO.

**Methods:** Eight patients who received venoarterial ECMO (V-A ECMO) were included in our study. All of them accepted continuous CrSO_2_ monitoring by near-infrared spectroscopy (NIRS) within 12 h of ECMO initiation until ECMO wean. Differences in the CrSO_2_ distribution characteristic, the variation trend of daily CrSO_2_, and the variability of CrSO_2_ for the first 5 days following ECMO initiation were compared between survivors and non-survivors according to pediatric intensive care unit (PICU) mortality.

**Results:** The percentage of time of CrSO_2_ <60% against the whole monitoring time was significantly lower in survivors in both hemispheres {right: 4.34% [interquartile range (IQR) = 0.39–8.55%] vs. 47.45% [IQR = 36.03–64.52%], *p* = 0.036; left: 0.40% [IQR = 0.01–1.15%] vs. 30.9% [IQR = 26.92–49.62%], *p* = 0.036}. Survivors had significantly higher CrSO_2_ on the first 4 days. Root mean of successive squared differences (RMSSD), the variability variable of CrSO_2_, was significantly lower in survivors (right: 3.29 ± 0.79 vs. 6.16 ± 0.67, *p* = 0.002; left: 3.56 ± 1.20 vs. 6.04 ± 1.44, *p* = 0.039).

**Conclusion:** Lower CrSO_2_, CrSO_2_ <60% over a longer period of time, and higher fluctuation of CrSO_2_ are likely associated with PICU mortality in pediatric patients undergoing V-A ECMO.

**Clinical Trial Registry:** URL: http://www.chictr.org.cn/showproj.aspx?proj=46639, trial registry number: ChiCTR1900028021.

## Introduction

Extracorporeal membrane oxygenation (ECMO) is an extracorporeal life support technology used for patients with cardiopulmonary failure as a severe refractory to conventional treatment measure (1–3). With the development of ECMO, it has been widely applied to the clinical (4–6); however, monitoring technology for ECMO support, particularly the brain function monitoring, has been developing quite slowly.

Near-infrared spectroscopy (NIRS) can provide useful data on cerebral tissue regional oxygen saturation (CrSO_2_). Because of its characteristics, such as non-invasiveness, continuousness, and being performed in real time and at the bedside (7–10), NIRS has been suggested as one of the regular monitoring technologies during ECMO support by the Extracorporeal Life Support Organization (ELSO) (11). Although NIRS is extensively used in various fields, there have been only a few studies on NIRS in the pediatric patients with ECMO. Consequently, the aim of this study was to explore the data distribution, variation trend, and variability of CrSO_2_ in pediatric patients undergoing ECMO so as to provide a relevant reference.

## Methods

### Patients

This prospective observational study was conducted between May 24, 2019, and January 18, 2020 in the 32-bed pediatric intensive care unit (PICU) of the Children's Hospital of Chongqing Medical University, China. All patients aged from 28 days to 18 years needing venoarterial ECMO (V-A ECMO) support were included in this study. Exclusion criteria included the following: with open heart surgery or cardiac catheterization procedure; with ECMO support <24 h; with validated cerebral injury before ECMO, including cerebral hemorrhage and ischemia; with serum total bilirubin >7 mg/dL before ECMO (12); with forehead skin damage not fit for NIRS monitoring.

Assessments of neurologic status were mainly based on the medical history and clinical symptoms and signs before ECMO. Patients suspected of having brain injury were examined with brain computed tomography (CT) or magnetic resonance imaging (MRI). Those patients without suspected brain injury and without abnormal neurologic imaging findings were all considered as without brain injury.

This study has been approved by the Institutional Review Board of Children's Hospital of Chongqing Medical University (2019-237) and has been performed in accordance with the ethical standards as laid down in the 1964 Declaration of Helsinki and its later amendments or comparable ethical standards and has been registered in Chinese Clinical Trial Registry (ChiCTR1900028021). Written informed consent for participation was obtained from children's parents or legal guardians.

### Data Collection

Baseline information before ECMO initiation was recorded and included: age, gender, weight, diagnosis, the interval of hospital admission to ECMO initiation, the interval of PICU admission to ECMO initiation, the interval of invasive mechanical ventilation begun to ECMO initiation, the use of vasoactive drugs before ECMO, PaO_2_/FiO_2_ ratio, and arterial blood gas parameters (pH, Paco_2_, Pao_2_, Sao_2_, lactate) before ECMO.

Follow-up variables included ECMO duration, ECMO parameters, treatment measures during ECMO, hemodynamic parameters during ECMO, duration of the invasive mechanical ventilation after ECMO wean, length of PICU and hospital stays, and PICU mortality.

### ECMO Management

All patients receiving ECMO support were managed by our ECMO team. We chose the mode of ECMO support in pediatric respiratory failure according to the patient's hemodynamic status and weight. We have to choose V-A ECMO in infants and low-weight toddlers with respiratory failure purely for a lack of double-lumen venovenous (V-V) cannulas in China. Venoarterial ECMO was surgically implanted using heparin-coated cannulation (Bio-Medicus, Medtronic Inc., Minneapolis, MN, USA) via the right internal jugular vein and the right common carotid artery. The centrifugal pump (Deltastream DP3, Medos, Stolberg, Germany) and membrane oxygenator (Hilite 800 LT, Hilite 2400 LT, or Hilite 7000 LT, Medos, Stolberg, Germany) were used. ECMO-related parameters and hemodynamic parameters were recorded every 2 h during ECMO support.

### Cerebral Tissue Regional Oxygen Saturation Monitoring

Monitoring of CrSO_2_ began within 12 h of ECMO initiation and continued until the weaning of ECMO using NIRS (FORE-SIGHT P/N01-06-2030C, CAS Medical Systems Inc., Branford, CT, USA). Cerebral tissue regional oxygen saturation data were gathered every 2 s and recorded on the device's memory. Our ECMO team did not adjust therapies following CrSO_2_ data. The daily median CrSO_2_ values were calculated for the first 5 days following ECMO initiation in both hemispheres and were then used to analyze the variation trend of CrSO_2_. At the same time, the median CrSO_2_ values over 4-h time intervals were calculated for the first 5 days and plotted as a time series. The variability of CrSO_2_ was evaluated using the standard deviation (SD), coefficient of variation (CV), and root mean of successive squared differences (RMSSD).

$$\begin{array}{l} RMSSD=\sqrt{\frac{\sum_{i=2}^{n}{\left(x_{i}-x_{i-1}\right)}^{2}}{n}} \end{array}$$

where *x*_*i*_ is the median CrSO_2_ at interval i (13). The differences of different CrSO_2_ variables were compared between survivors and non-survivors.

### Outcome

The primary outcome was PICU mortality. All patients weaned off ECMO successfully had accepted brain CT or MRI as soon as the patients' condition relatively stabilized.

### Statistical Analysis

Statistical analysis was performed with SPSS version 26 (IBM Corp., Armonk, NY, USA). Continuous variables were confirmed for normal distribution using the Shapiro–Wilk test and are presented either as means ± SD for data with a normal distribution or as median (IQR) for non-normal data. The differences between groups were tested with Student *t*-test, Satterthwaite *t*-test, or Mann–Whitney *U*-test, as appropriate. Categorical variables are presented as counts (percentages). Repeated measurement data were analyzed using the generalized estimating equation. *p* < 0.05 was considered as statistically significant.

## Results

### Study Population

Thirteen patients accepted ECMO support during the study period. Five of them were excluded from the analysis: two had V-V ECMO for respiratory failure, one died within 24 h of ECMO initiation, and two accepted V-A ECMO following open heart surgery. Finally, eight patients undergoing V-A ECMO were included in the study. Patients' demographics and clinical characteristics are summarized in Table 1. Six patients weaned off ECMO successfully, whereas one of them could not detach from the ventilator after ECMO and died of respiratory failure. Two patients had no improvement and died after decannulation. Two patients had abnormal neurologic imaging findings. Patient 4 had intracranial hemorrhage, and patient 8 had cerebral infarction, whereas both of them survived to PICU discharge. For all eight included patients, the PICU length of stay was 20 ± 7 days, and the hospital length of stay was 45 ± 24 days. Arterial blood gas parameters before ECMO were pH 7.40 ± 0.10, Paco_2_ = 44.4 ± 7.5 mm Hg, Pao_2_ = 47.4 ± 13.0 mm Hg, Sao_2_ = 79.6 ± 12.5%, and lactate = 1.6 ± 0.9 mmol/L. PaO_2_/FiO_2_ ratio before ECMO was 48 ± 14.

**Table 1:** Patients' demographics and clinical characteristics.

**Case**	**Age, months**	**Weight, kg**	**Sex**	**Diagnosis**	**Hospital-ECMO interval,^a^ days**	**PICU-ECMO interval,^b^ days**	**MV-ECMO interval,^c^ days**	**ECMO duration, hours**	**VIS-max before ECMO^d^**	**ECMO wean**	**MV-post, hours**	**PICU LOS, days**	**Hospital LOS, days**	**PICU survival**
1	4	8.0	M	HAdV pneumonia, PARDS	9	8	8	118	5	Yes	141	21	50	Yes
2	84	24.0	F	HAdV pneumonia, PARDS	19	4	4	116	0	Yes	163	21	62	Yes
3	47	15.0	F	HAdV pneumonia	3	2	2	115	26	Yes	43	11	19	Yes
4	10	10.5	M	HAdV pneumonia, PARDS	7	7	7	145	10	Yes	136	32	93	Yes
5	35	11.0	F	HAdV pneumonia, PARDS	18	18	18	163	10	No	—	27	29	No
6	23	9.5	M	HAdV pneumonia, PARDS	20	3	3	243	0	No	—	14	30	No
7	1	3.4	F	HAdV pneumonia, PARDS	16	1	13	283	0	Yes	165	19	34	No
8	24	11.0	M	Bilateral PC	1	1	1	41	32	Yes	115	14	41	Yes

*ECMO, extracorporeal membrane oxygenation; PICU, pediatric intensive care unit; MV, mechanical ventilation; VIS-max, the maximum of the vasoactive–inotropic score; MV-post, duration of the invasive mechanical ventilation after ECMO wean; PICU LOS, PICU length of stay; hospital LOS, hospital length of stay; M, male; F, female; HAdV, human adenovirus; PARDS, pediatric acute respiratory distress syndrome; PC, pulmonary contusion*.

a *The interval of hospital admission to ECMO initiation*.

b *The interval of PICU admission to ECMO initiation*.

c *The interval of invasive mechanical ventilation begin to ECMO initiation*.

d *VIS = epinephrine (μg/kg per min) × 100 + norepinephrine (μg/kg per min) × 100 + milrinone (μg/kg per min) × 10 + vasopressin (U/kg per min) × 10,000 + dopamine (μg/kg per min) + dobutamine (μg/kg per min) (14, 15)*.

### ECMO Management

The centrifugal pump rotating speed was 5,468 ± 347 r/min, and the ECMO flow was 70 ± 10 mL/kg/min for the first 5 days following ECMO initiation. Patients' mean arterial pressure (MAP) was 71 ± 12 mm Hg, SvO_2_ was 66 ± 3%, and hematocrit was 35 ± 2%. All patients accepted vasoactive drugs during ECMO support, and the duration of vasoactive drugs use was 1.5 days (IQR = 1–3 days). The maximum vasoactive–inotropic score (14, 15) was 17.85 (IQR = 5.36–31.38). Three (37.5%) patients received continuous renal replacement therapy during ECMO support, and all of them suffered PICU mortality.

### Cerebral Tissue Regional Oxygen Saturation Variables

**Figure 1:**
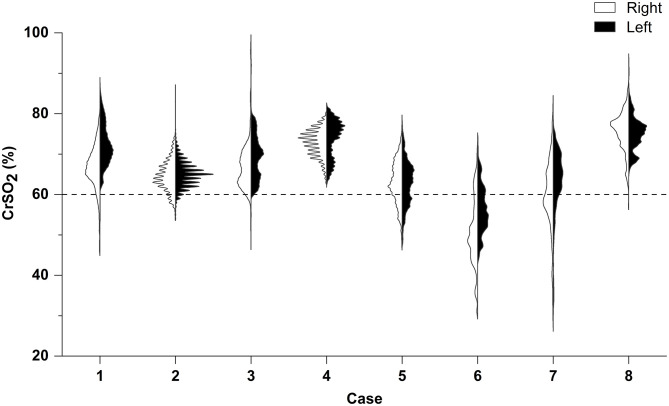
Right–left split violin plot of CrSO_2_ for the first 5 days following ECMO initiation. According to PICU mortality, patients 5, 6, and 7 suffered death; others survived. Results of the Mann–Whitney *U*-test indicate that the percentage of time of CrSO_2_ <60% against the whole monitoring time was significantly lower in survivors in both hemispheres (both *p* < 0.05).

The duration of CrSO_2_ monitoring was 79.0 ± 17.0% of the time on the first 5 days of ECMO. The distribution of CrSO_2_ data for the first 5 days in both hemispheres is shown in Figure 1. The percentage of time of CrSO_2_ <60% against the whole monitoring time was significantly lower in survivors in both hemispheres [right: 4.34% (IQR = 0.39–8.55%) vs. 47.45% (IQR = 36.03–64.52%), *p* = 0.036; left: 0.40% (IQR = 0.01–1.15%) vs. 30.9% (IQR = 26.92–49.62%), *p* = 0.036)].

**Figure 2:**
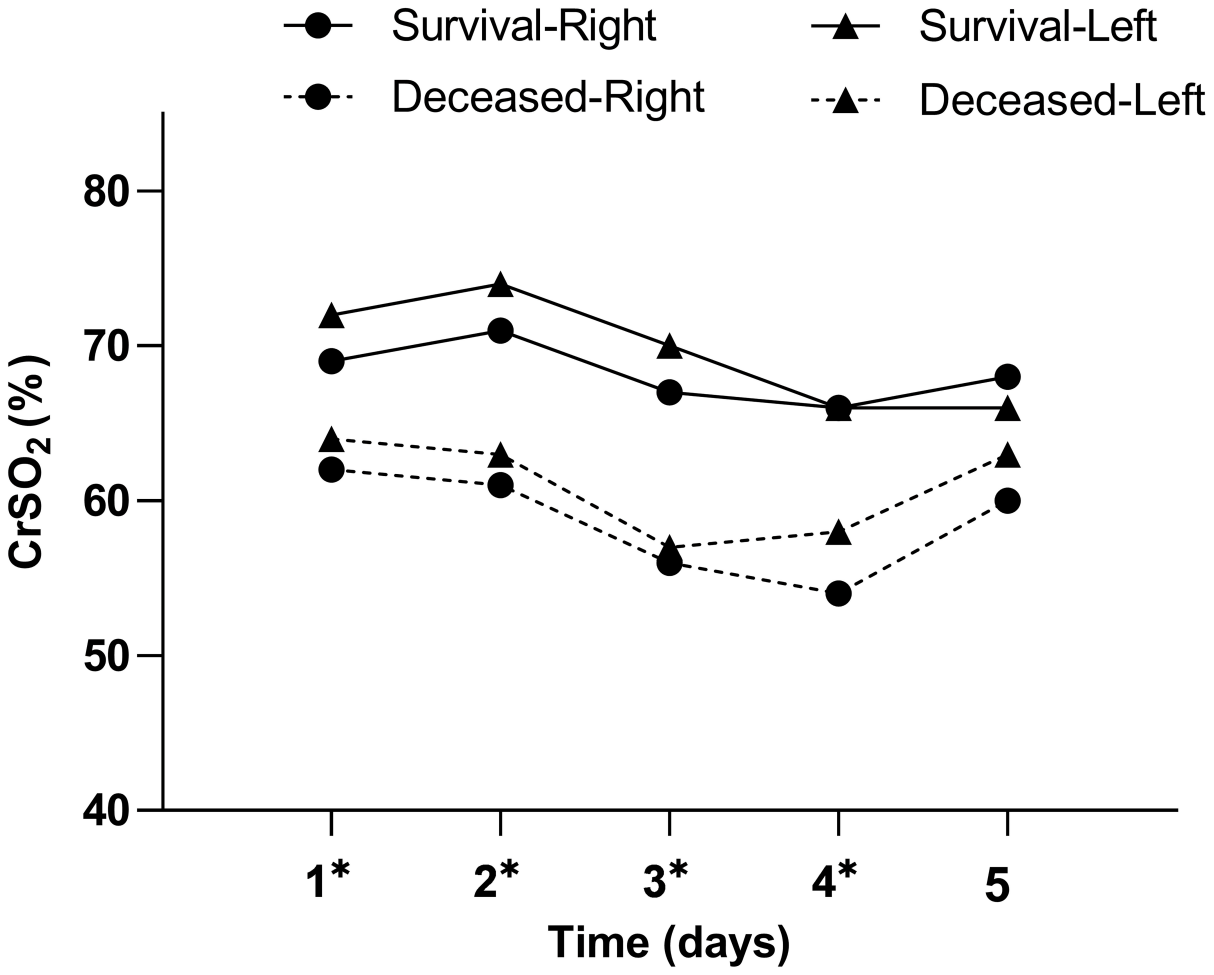
Daily CrSO_2_ for the first 5 days following ECMO initiation according to PICU mortality. Results of the generalized estimating equation (GEE) indicate significant differences between survivors and non-survivors for each of the first 4 days following ECMO initiation in both hemispheres. *p < 0.05.

Table 2 and Figure 2 show CrSO_2_ variation trends for the first 5 days in both hemispheres. Because of the risk of developing pressure sores, we did not gather CrSO_2_ signals in the fifth day for patient 1. While the duration of ECMO support was only 41 h for patient 8; thus, the patient did not contribute data to the third, fourth, and fifth day. Survivors had significantly higher CrSO_2_ for each of the first 4 days in both hemispheres (all *p* < 0.05), but there were no significant differences between survivors and non-survivors on the fifth day in both hemispheres (right: Wald χ^2^ = 3.402, *p* = 0.065, left: Wald χ^2^ = 1.216, *p* = 0.270).

**Table 2:** CrSO_2_ for the first 5 days following ECMO initiation according to PICU mortality.

	**Group**		**Time**					**Sum**	**Wald χ^**2**^^a^**	***p-*Value**
			**Day 1**	**Day 2**	**Day 3**	**Day 4**	**Day 5**			
Right CrSO_2_	Survival	Mean	69	71	67	66	68	68	24.942	**<0.001**
		SD	3	5	6	3	6	5		
	Deceased	Mean	62	61	56	54	60	59	24.430	**<0.001**
		SD	4	7	8	8	10	7		
	Sum	Mean	66	67	62	61	64	64	191.856	**<0.001**
		SD	5	7	9	8	9	7		
	Wald χ^2^		8.633	5.730	8.173	10.950	3.402	8.803	31.814	**<0.001**
	*p-*Value		**0.003**	**0.017**	**0.004**	**0.001**	0.065	**0.003**		
Left CrSO_2_	Survival	Mean	72	74	70	66	66	70	199.500	**<0.001**
		SD	4	6	4	3	2	5		
	Deceased	Mean	64	63	57	58	63	61	684.628	**<0.001**
		SD	1	6	2	7	6	5		
	Sum	Mean	69	70	65	63	65	66	110.333	**<0.001**
		SD	5	8	8	6	4	7		
	Wald χ^2^		22.405	7.344	59.748	6.485	1.216	13.628	61.187	**<0.001**
	*p-*Value		**<0.001**	**0.007**	**<0.001**	**0.011**	0.270	**<0.001**		

*p < 0.05 was considered significant. Results of the GEE indicated that there were significant differences between survivors and non-survivors for the first 4 days following ECMO initiation in both hemispheres. There was a significant interactive effect of ECMO support time and treatment effect on CrSO_2_ in both hemispheres*.

*Significant p-values are indicated in bold type in the table*.

*CrSO_2_, cerebral tissue regional oxygenation saturation; GEE, generalized estimating equation*.

a *Wald χ^2^ is the test statistics of the GEE*.

**Figure 3:**
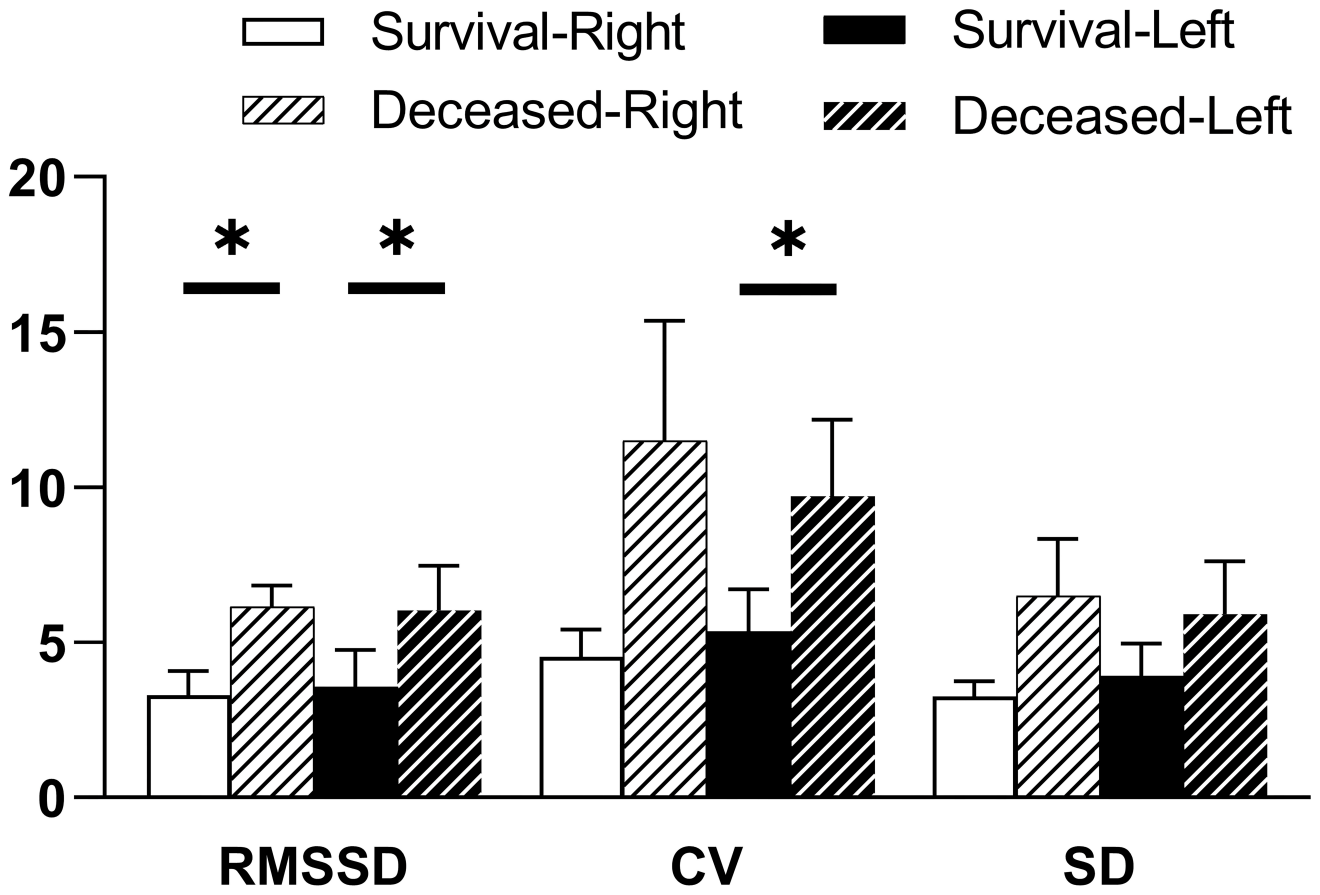
CrSO_2_ variability for the first 5 days following ECMO initiation according to PICU mortality. Indicators of CrSO_2_ variability are RMSSD, root mean of successive squared differences; CV, coefficient of variation; SD, standard deviation. Results of the Student *t*-test indicate significant differences between survivors and non-survivors for right RMSSD, left RMSSD, and left CV, respectively. *p < 0.05.

The indicators of CrSO_2_ variability between survivors and non-survivors for the first 5 days following ECMO initiation are shown in Figure 3. Root mean of successive squared differences (RMSSDs) were significantly lower in survivors in both hemispheres (right: 3.29 ± 0.79 vs. 6.16 ± 0.67, *p* = 0.002; left: 3.56 ± 1.20 vs. 6.04 ± 1.44, *p* = 0.039). Coefficient of variation was significantly lower in survivors only in the left hemisphere (right: 4.54 ± 0.89 vs. 11.50 ± 3.85, *p* = 0.084; left: 5.36 ± 1.36 vs. 9.71 ± 2.47, *p* = 0.016). Standard deviation was seemingly lower in survivors in both hemispheres, but there were no significant differences (right: 3.27 ± 0.47 vs. 6.50 ± 1.83, *p* = 0.087; left: 3.92 ± 1.03 vs. 5.91 ± 1.71, *p* = 0.081).

## Discussion

Monitoring technology has not been developed along with ECMO-related technologies, and monitoring of the central nervous system is particularly challenging. The head ultrasound was used in patients undergoing ECMO with several limitations (16, 17), for example, this approach could not capture the neurologic imaging in pediatric patients with closed cranial fontanels and adult patients (8). Besides, it cannot be used for continuous monitoring. As for electroencephalography, amplitude-integrated electroencephalography, and somatosensory-evoked potentials, a few studies have tested their roles in ECMO support (8, 18). Although head CT and MRI can clearly detect the cerebral hemorrhage or ischemia, the hospital transfer may have potential risks (8). Different from all these monitoring methods, cerebral NIRS can provide a continuous value of CrSO_2_. Because of its non-invasiveness, continuousness, and being performed in real time and at the bedside (7–10), ELSO suggests regular application of NIRS to patients undergoing ECMO support (11). Nonetheless, there was not a certain method to use NIRS. In our study, we explored the CrSO_2_ distribution characteristic, the variation trend of daily CrSO_2_, and the variability of CrSO_2_ for the first 5 days following ECMO initiation.

In the present study, survivors had higher CrSO_2_ for the first 4 days following ECMO initiation in both hemispheres, and the percentage of time of CrSO_2_ <60% against the whole monitoring time was lower in survivors. Previous studies also revealed that survivors had higher CrSO_2_ than non-survivors (19, 20). And the occurrence of cerebral desaturation, which is defined as CrSO_2_ <60% for >5% of the entire monitoring period, was independently associated with hospital mortality (21). Wong et al. (22) adjusted therapy measures that could affect the cerebral oxygen balance to improve CrSO_2_ values. They also found that unilateral or bilateral CrSO_2_ decreasing persistently was associated with worse outcome. We have similar findings and consider that lower CrSO_2_ and the longer time of CrSO_2_ <60% reduce the possibility of survival. However, there was no significant difference for the median CrSO_2_ of the fifth day among survivors and non-survivors. We guess volume overload may play a role in this phenomenon, because all deceased patients in our study accepted continuous renal replace therapy in the later days of ECMO support. Studies suggested cerebral autoregulation impairment during ECMO was associated with worse outcome (20, 23). Excessive blood would stream into brain circulation without the regulation of cerebral autoregulation, so that the CrSO_2_ signal gets higher.

Besides, for the indicators of CrSO_2_ variability, bilateral RMSSD and left CV were significantly higher in non-survivors; meanwhile, bilateral SD and right CV also showed the trend to be higher in non-survivors. Therefore, we speculate that greater fluctuation of CrSO_2_ is associated with a worse prognosis. Absolute CrSO_2_ values can be different between different types of NIRS devices, because of different algorithms (24). Different from using absolute CrSO_2_ value directly, indicators of CrSO_2_ variability can reduce the error caused by different types of NIRS devices. Using RMSSD to describe the variability of CrSO_2_ had been studied in perioperative period of cardiac surgery. Spaeder et al. (13) found that lower RMSSD was associated with poor neurodevelopmental outcomes in neonatal survivors of congenital heart disease. Meanwhile, Flechet et al. (25) found that pediatric patients with lower RMSSD had larger probability of developing acute kidney injury after cardiac surgery. This is the first study to explore the CrSO_2_ variability using RMSSD in patients with ECMO support. However, different from those findings that came from the perioperative period of cardiac surgery, survivors had a lower RMSSD in our study. We consider that there are several potential factors that may cause this difference. First, patients with open heart surgery were excluded in our study, whereas those two previous studies had focused on the perioperative period of cardiac surgery. Second, conclusion of those two previous studies came from CrSO_2_ data gathered from postoperative period. Cerebral blood flow is pulsatile for most patients in this period, whereas cerebral blood flow in V-A ECMO is non-pulsatile or partial pulsatile, which is associated with endothelial dysfunction, decreased microvascular perfusion, and increased vascular resistance (26). Third, the total monitoring duration is longer in our study. Meanwhile, those two previous studies averaged CrSO_2_ over 1-min intervals to calculate RMSSD. But we calculated RMSSD using the median CrSO_2_ over 4-h intervals, because missing data cannot be avoided in such long a time in clinical. We have checked CrSO_2_ signal every 4–6 h, so that we have enough data in every 4-h intervals. Further studies are needed to confirm our findings and explore underlying mechanisms.

There are some limitations to our study. First, our findings came from a small sample size and single-center study, and most patients in our study had a similar diagnosis of human adenovirus pneumonia. Those make it cautious to interpret the results of our study. Second, only patients receiving V-A ECMO were included in our study; thus, conclusions obtained from the current study may not be extrapolated to those with V-V ECMO. However, Clair et al. (19) reported the mean CrSO_2_ for the whole time of ECMO support, the duration of CrSO_2_ below the threshold of 20% from baseline, and the duration of CrSO_2_ <50% were not different in patients with V-V ECMO compared to V-A ECMO. Third, we only compared CrSO_2_ values between survivors and non-survivors, as the small sample size is a limitation in adjusting for suspected confounding factors. The relationships between CrSO_2_ values and hemodynamic parameters were not discussed. At the same time, we did not explore the correlation between CrSO_2_ and neurologic complications. Studies found that lower CrSO_2_ and more frequently occurrence of cerebral desaturation in patients with ECMO were more likely related to brain injury (19, 27, 28). However, different studies had different definitions of cerebral desaturation and brain injury. Tian et al. (29) assessed the dynamic relationship between the MAP and CrSO_2_ fluctuations and found that patients with significant in-phase MAP to CrSO_2_ coherence always had abnormal neurologic imaging findings. Finally, there are limitations in CrSO_2_ monitoring. CrSO_2_ mainly represents the regional oxygen saturation of the forehead. Thus, it may be hard to evaluate the deeper part or other region of the brain. Meanwhile, CrSO_2_ can be affected by serum bilirubin, skin surface temperature, or scalp blood flow (12, 21, 28).

## Conclusion

Cerebral tissue regional oxygen saturation can be used as a regular monitoring parameter for pediatric patients undergoing ECMO. Lower CrSO_2_, CrSO_2_ <60% over a longer period of time, and higher fluctuation of CrSO_2_ are likely associated with PICU mortality in pediatric patients undergoing V-A ECMO. Future multicenter and large-scale studies are needed to mitigate confounding factors and verify our findings and explore the correlation between neurologic complications and CrSO_2_ variables in our study.

## Data Availability Statement

The raw data supporting the conclusions of this article will be made available by the authors, without undue reservation.

## Ethics Statement

The studies involving human participants were reviewed and approved by Institutional Review Board of Children's Hospital of Chongqing Medical University. Written informed consent to participate in this study was provided by the participants' legal guardian/next of kin.

## Author Contributions

SC is the principal author and contributed to data collection, data analysis and interpretation, and drafted the manuscript. SC and FX contributed to the study conception and design. FF, WL, CL, and FX contributed to data analysis and interpretation and revised the manuscript critically for important intellectual content. All authors contributed to the article and approved the submitted version.

## Conflict of Interest

The authors declare that the research was conducted in the absence of any commercial or financial relationships that could be construed as a potential conflict of interest.

## Abbreviations

ECMO: extracorporeal membrane oxygenation
V-A ECMO: venoarterial ECMO
V-V ECMO: venovenous ECMO
PICU: pediatric intensive care unit
NIRS: near-infrared spectroscopy
CrSO_2_: cerebral tissue regional oxygen saturation
SD: standard deviation
CV: coefficient of variation
RMSSD: root mean of successive squared differences
GEE: the generalized estimating equation.
